# Zinc Oxide Nanoparticle-Mediated Root Metabolic Reprogramming for Arsenic Tolerance in Soybean

**DOI:** 10.3390/plants13223142

**Published:** 2024-11-08

**Authors:** Muhammad Zeeshan, Anas Iqbal, Abdul Salam, Yuxin Hu, Aamir Hamid Khan, Xin Wang, Xiaoran Miao, Xiaoyuan Chen, Zhixiang Zhang, Peiwen Zhang

**Affiliations:** 1 State Key Laboratory of Green Pesticide, South China Agricultural University, Guangzhou 510642, China; 11616102@zju.edu.cn (M.Z.);; 2 Yingdong College of Biology and Agriculture, Shaoguan University, Shaoguan 512005, China; 3 Guangzhou Key Laboratory for Science and Technology of Fragrant Rice, Guangzhou 510642, China; anasiqbal@scau.edu.cn; 4 College of Pastoral Agriculture Science and Technology, Lanzhou University, Lanzhou 730000, China; hyxjyo@163.com; 5 Department of Biogeography, Paleoecology and Nature conservation, Faculty of Biology and Environmental Protection, University of Lodz, 90-237 Lodz, Poland; aamir.hamid.khan@biol.uni.lodz.pl

**Keywords:** amino acid metabolism, arsenate tolerance, *Glycine max* L, metabolome, metabolic pathways, TCA cycle, engineered NP

## Abstract

Arsenate (AsV) is absorbed and accumulated by plants, which can affect their physiological activities, disrupt gene expression, alter metabolite content, and influence growth. Despite the potential of zinc oxide nanoparticles (ZnONPs) to mitigate the adverse effects of arsenic stress in plants, the underlying mechanisms of ZnONPs-mediated detoxification of AsV, as well as the specific metabolites and metabolic pathways involved, remain largely unexplored. In this study, we demonstrated root metabolomic profiling of soybean germinating seedlings subjected to 25 μmol L^−1^ arsenate (Na_2_HAsO_4_) and ZnONPs at concentrations of 25 μmol L^−1^ (ZnO25) and 50 μmol L^−1^ (ZnO50). The objective of this study was to examine the effects on soybean root metabolomics under AsV toxicity. Metabolomic analysis indicated that 453, 501, and 460 metabolites were significantly regulated in response to AsV, ZnO25, and ZnO50 treatments, respectively, compared to the control. Pathway analysis of the differentially regulated metabolites (DRMs) revealed that the tricarboxylic acid (TCA) cycle, glutathione metabolism, proline and aldarate metabolism, and arginine and proline metabolism were the most statistically enriched pathways in ZnONPs-supplemented plants. These findings suggest that ZnONPs enhance the tolerance response to AsV. Collectively, our results support the hypothesis that ZnONPs fertilization could be a potential strategy for improving soybean crop resilience under AsV stress.

## 1. Introduction

Over the past few decades, anthropogenic and industrial activities have significantly contributed to the release of heavy metals and potentially toxic elements into the environment, including agricultural soils. Arsenic (As), due to its persistence in soil and frequent presence in various sources of contamination, is considered one of the most significant environmental contaminants [1]. In the environment, As exists in multiple chemical forms, with inorganic AsV and arsenite (AsIII) being the most prevalent. In surface soils, AsV is predominantly found, whereas AsIII is more common in anaerobic conditions [2]. When AsV is absorbed and accumulated by plants, it disrupts various physiological and biological processes, including the accumulation of cellular toxins, damage to membrane integrity, a reduction in osmolytes, growth inhibition, and impairment of the photosynthetic apparatus [3]. According to Thakur et al. [4], AsV stress significantly downgrades the plant hormone signal transduction, amino acid metabolism, glutathione metabolism, and carbon metabolism pathways in Indian mustard, leading to a reduced stress response. In recent years, research on As has primarily focused on its uptake, accumulation, and transformation, as well as its effects on plant and human health. AsV is chemically analogous to inorganic phosphate (Pi) ions and is therefore taken up by plant roots via the Pi transporters system [5]. Additional significant areas of study encompass As mobilization, uptake mechanisms, and the advancement of detection methods and remediation techniques [6]. 

Numerous studies have demonstrated that engineered nanoparticles (ENPs) can significantly reduce the accumulation of potentially toxic elements in plants and/or soil systems, including As, copper (Cu), cadmium (Cd), and lead (Pb), among others [7,8,9,10]. Research has indicated that the impact of these NPs on plant growth can be both beneficial and detrimental, varying with plant species, NP concentration, size, and surface area [11,12,13,14,15]. ZnONPs, one of the most frequently utilized ENPs in plant–soil systems, are applied to mitigate abiotic stressors, including the analysis of physiological, biochemical, and transcriptomic responses [13]. Investigations have confirmed that ZnONPs, when used in optimal concentration, can enhance photosynthetic carbon assimilation and decrease the accumulation of potentially toxic elements in various plant species [9,10,16]. In contrast, studies have found that high concentrations and large-size ZnONPs negatively affect soybean growth and development [17,18]. Nevertheless, the potential metabolites and metabolic pathways responsible for arsenic tolerance in soybean under ZnONPs exposure remain unexplored.

Metabolomics, a multidisciplinary field, is utilized in toxicological studies to enhance our comprehension of metabolic responses to toxicants through contemporary analytical techniques [19]. These techniques enable the assessment of metabolite levels, which serve as proxies for plant responses to both genetic and environmental perturbations [20]. Quantitative analysis of metabolite concentrations under stress conditions offers precise insights into the molecular mechanisms underlying plant tolerance. Plants modulate a range of metabolic pathways to mitigate damage and sustain homeostasis in environments contaminated with heavy metals [21]. Consequently, metabolomic analysis is a suitable approach to enhance our understanding of plant responses to potential toxic elements [22]. Recent research has identified metabolic pathways involving amino acids and their derivatives, carbohydrates, and organic acids in the arsenic stress response of the hyper-accumulator *P. vittata*, as revealed by liquid chromatography–mass spectrometry/mass spectrometry (LC-MS/MS)-based metabolomic analysis [23]. Wild soybean has also been found to contain higher levels of metabolites related to acetylated amino acids, sugar alcohols, and disaccharides compared to cultivated soybean but lower quantities of unsaturated fatty acids, carboxylic acids, and monosaccharides that mediate salt stress [24]. Similarly, Zhu et al. [25] reported that pathways such as arginine and proline metabolism, phenylpropanoid biosynthesis, flavone and flavonol biosynthesis, and nitrogen metabolism are crucial in alleviating Cd toxicity in tall fescue. Primary metabolites in plants, including amino acids, enzymes, and carbohydrates, are essential for optimal plant growth processes and for facilitating growth and development [26,27]. Plants respond to stress by altering mRNA expression, protein regulation, and metabolite accumulation at the molecular level, all of which are considered defense mechanisms [28].

In the present investigation, we hypothesized that specific metabolites, crucial for regulating essential metabolic pathways, could play a significant role in soybean tolerance to AsV. To validate this hypothesis, we employed an untargeted metabolomics strategy involving liquid chromatography–mass spectrometry (LC/MS) to identify differential metabolites in soybean roots subjected to AsV toxicity, with and without the supplementation of ZnONPs. The objective of this analysis was to delineate the metabolic pathways involved in the modulation of AsV tolerance. The findings offer novel insights into the mechanisms of arsenic accumulation and detoxification in soybeans, potentially enhancing our understanding of arsenic tolerance mechanisms.

## 2. Results

### 2.1. QC and Multivariate Analysis Suggest a Highly Variable and Reliable Data Set and Metabolites Reprogramming in Soybean 

**Figure 1:**
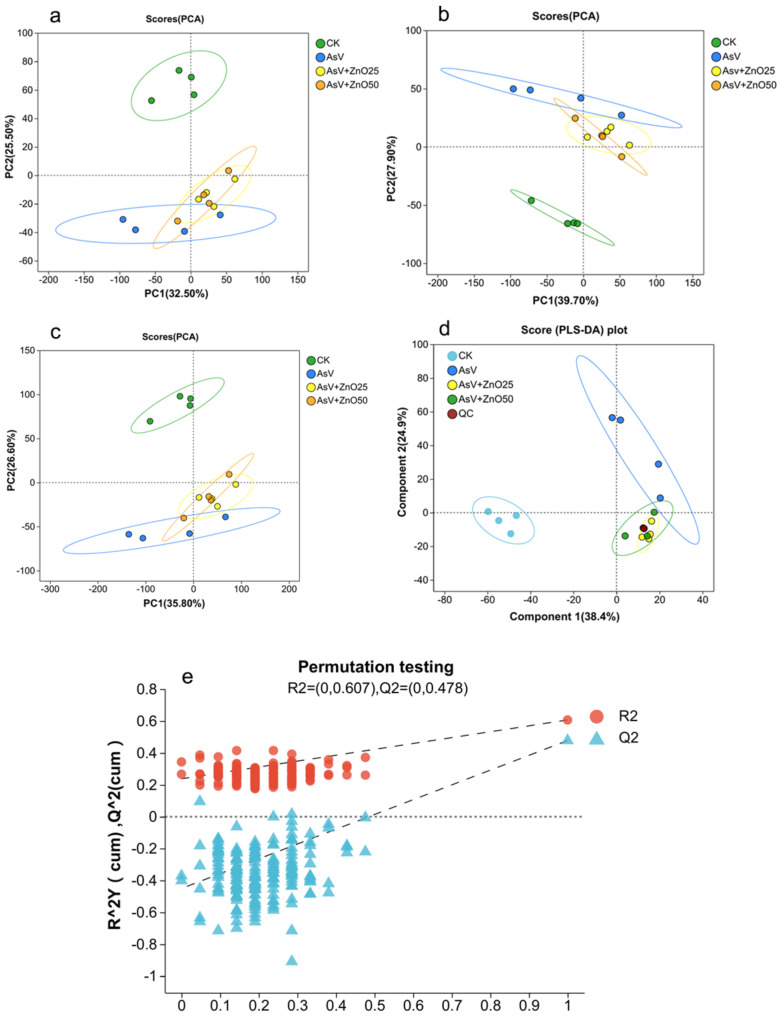
Two-dimensional principal component analysis (PCA) for the positive ion mode (**a**), negative ion mode (**b**), and a combined analysis of both modes (**c**). Partial least squares discriminant analysis (PLS-DA) score plots (**d**) and a permutation test (**e**) for the liquid chromatography–mass spectrometry (LC-MS) metabolic profiles of soybean roots subjected to treatments with 25 µmol L^−1^ AsV and ZnONPs at concentrations of 25 μmol L^−1^ (2.035 mg L^−1^) and 50 µmol L^−1^ (4.07 mg L^−1^). R2 and Q2 stand for model interpretability and model predictability respectively.

We employed liquid chromatography–mass spectrometry (LC–MS) to profile the metabolome of soybean roots under AsV alone and in combination with ZnONPs supplementation. Following data normalization and the exclusion of low-quality spectra, we detected 997 metabolites from 8600 positive ion peaks and 495 metabolites from 7427 negative ion peaks across four libraries (Table S1). We subjected the dataset to both univariate and multivariate analyses, including principal component analysis (PCA), partial least squares discriminant analysis (PLS-DA), and hierarchical cluster analysis (HCA) (Figure 1a–e and Figure S1). PCA, an unsupervised method, was used to identify patterns of separation among treatments and to identify outliers, thereby revealing the extent of variability within the dataset across and within different groups [29]. The PCA score plot indicated that PC1 explained 32.50% (R^2^ = 0.325) and PC2 explained 25.50% (R^2^ = 0.235) of the total variance in the positive ion mode, whereas in the negative ion mode, PC1 and PC2 accounted for 39.70% (R^2^ = 0.397) and 27.90% (R^2^ = 0.2.676), respectively, with no outliers detected (Figure 1a,b). The combined PCA of both ion modes demonstrated that metabolites were differentiated by treatment (Figure 1c). The control (CK) dataset was distinctly separated from the other three datasets (AsV, AsV + ZnO25, and AsV + ZnO50), and the replicates within each set clustered together, indicating significant differences in the metabolite profiles.

The partial least squares discriminant analysis (PLS-DA), a supervised statistical model designed for the analysis of large datasets, was used to establish the relationship between sample categories and metabolite expression [30,31]. By incorporating the variable importance in projection (VIP) score, PLS-DA aids in identifying metabolites with differential expression patterns between treatments, relying on their influence and explanatory power in sample group classification and discrimination [27,31]. Our results demonstrated a clear separation between the treated and control (CK) groups. A permutation test conducted over 200 iterations resulted in R2 = (0, 0.607) and Q2 = (0, 0.478), with a *p*-value < 0.05, confirming the absence of overfitting in the PLS-DA model (Figure 1d,e). Hierarchical cluster analysis (HCA) revealed substantial variation in metabolite content across the 16 samples, which were categorized into four distinct clusters, suggesting that metabolite production is treatment-specific (Figure S1). Collectively, these findings indicate that the dataset is highly variable and reliable, suitable for further analysis. Additionally, the dataset suggests that the treatment groups significantly reprogrammed the metabolite profile of soybean roots.

The analysis of the UpSet Venn diagram revealed that 1235, 1342, 1437, and 1427 metabolites were identified in the CK, AsV, AsV + ZnO25, and AsV + ZnO50 groups, respectively (Figure S2). Furthermore, 1169 metabolites were shared among all four groups, with 13 and 4 metabolites unique to the AsV + ZnO25 and AsV + ZnO50 treatments, respectively.

### 2.2. ZnONPs Modulated the Soybean Root Metabolome Under AsV Stress

**Figure 2:**
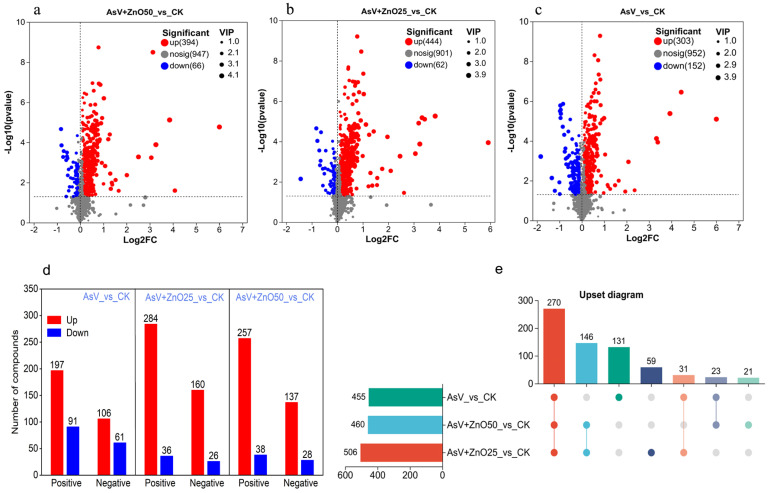
The metabolomics data from soybean roots treated with 25 μmol L^−1^ AsV and 25 μmol L^−1^ (2.034 mg L^−1^) and 50 μmol L^−1^ (4.07 μmol L^−1^) ZnONPs. The figure includes (**a**) a volcano plot of differentially regulated metabolites (DRMs) in the positive ion mode, (**b**) a volcano plot of DRMs in the negative ion mode, (**c**) a combined volcano plot of all DRMs, (**d**) a bar graph depicting the number of DRMs specifically regulated in either the positive or negative ion mode, and (**e**) an UpSet Venn diagram of DRMs. Each point in the plots represents a detected compound from the metabolomic dataset, with red and blue colors indicating up-regulated and down-regulated compounds, respectively, relative to the CK. The size of each point signifies the variable importance in projection (VIP) value.

The differential regulated metabolites (DRMs) were examined under AsV stress alone and following ZnONPs treatments, in comparison with the CK treatment, to elucidate metabolites closely associated with AsV tolerance in soybean roots. The screening criteria were established using a *t*-test *p*-value < 0.05 and a variable importance in the projection (VIP) value in OPLS-DA ≥ 1 to characterize the DRMs. The volcano plot revealed that 455 metabolites were significantly altered in the AsV_vs_CK treatment, with 303 up-regulated and 152 down-regulated (Figure 2a, Table S2). In the positive and negative modes, 197 and 106 metabolites were up-regulated, while 91 and 61 were down-regulated, respectively (Figure 2d). Under AsV + ZnO25 and AsV + ZnO50 treatments, 506 and 460 metabolites were significantly altered compared to the CK, with 444 and 394 up-regulated and 62 and 66 down-regulated, respectively (Figure 2b,c; Tables S3 and S4). Further data analysis indicated that under AsV + ZnO25, 284 and 160 metabolites were up-regulated, and 36 and 26 were down-regulated in the positive and negative modes, respectively. Similarly, under AsV + ZnO50, 257 and 137 metabolites were up-regulated, and 38 and 28 were down-regulated in the positive and negative modes, respectively (Figure 2d). Across all treatments, the number of up-regulated DRMs exceeded that of down-regulated DRMs. Notably, 131, 59, and 21 DRMs were uniquely regulated under AsV, AsV + ZnO25, and AsV + ZnO50 treatments, respectively, whereas 270 DRMs were commonly regulated across all three treatments (Figure 2e).

### 2.3. Hierarchical Cluster Analysis of Differentially Regulated Metabolites

**Figure 3:**
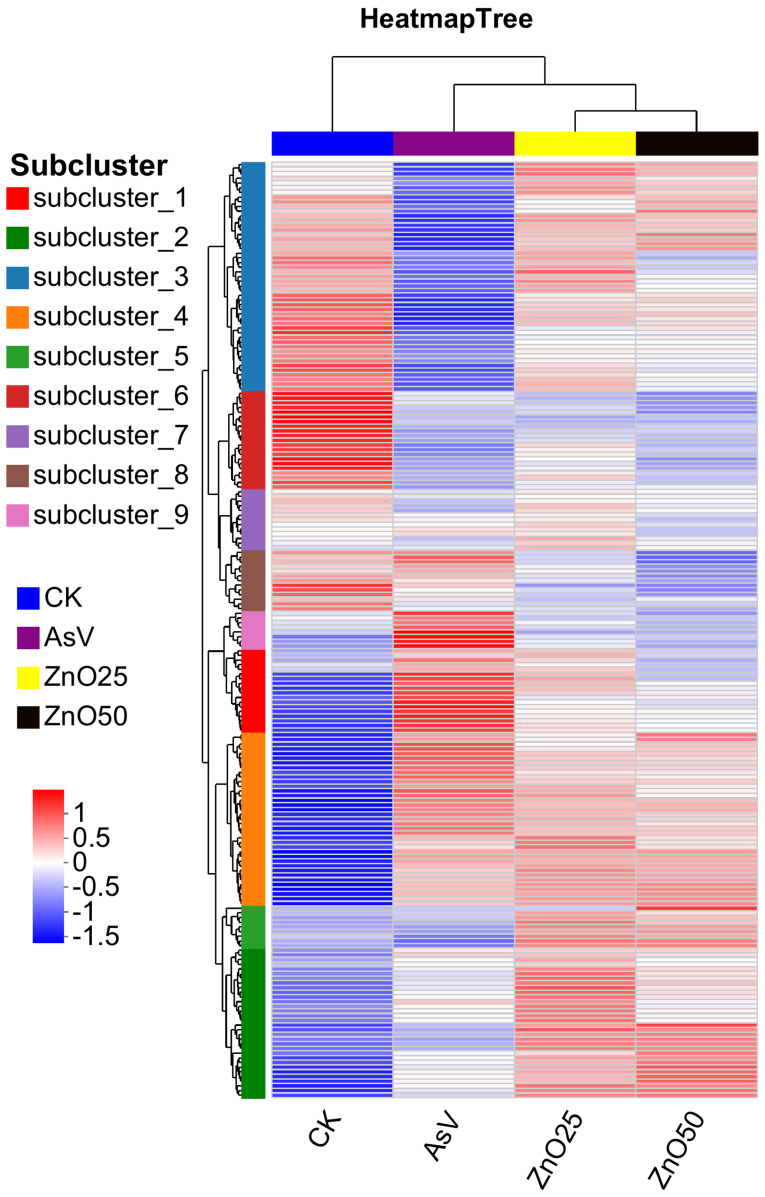
Hierarchical clustering heatmap depicting the top 200 metabolites across various treatment groups. Each column corresponds to a sample and each row to a metabolite, with color intensity reflecting the relative expression levels within the sample group. Concentrations are represented by normalized intensity values, where red signifies high abundance and blue indicates low abundance. The treatment concentration was 25 µmol L^−1^ AsV and ZnONPs at concentrations of 25 µmol L^−1^ (2.035 mg L^−1^) and 50 µmol L^−1^ (4.07 mg L^−1^).

**Figure 4:**
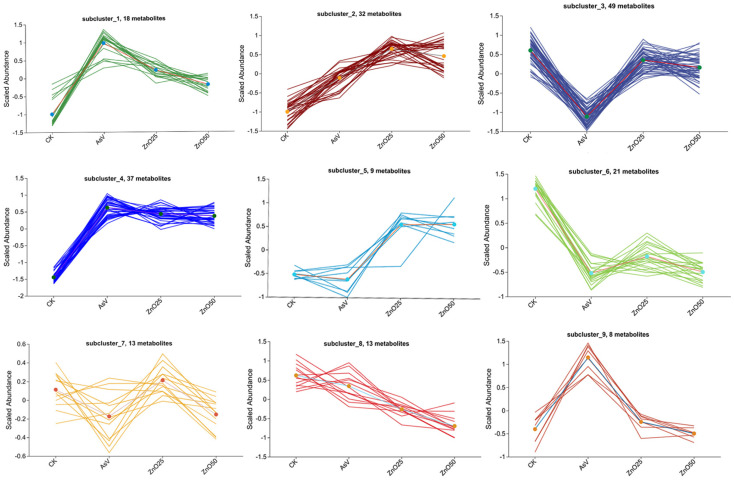
The *k*-means clustering analysis of the DAMs in the roots among different treatments. The treatment concentration was 25 µmol L^−1^ AsV and ZnONPs at concentrations of 25 µmol L^−1^ (2.035 mg L^−1^) and 50 µmol L^−1^ (4.07 mg L^−1^).

To elucidate the alterations and subsequent segregation of metabolites, a heatmap coupled with hierarchical clustering was employed. This approach identified the top 200 differentially regulated metabolites (Figure 3, Table S5). The heatmap delineated distinct clusters of metabolites across the four treatment groups, suggesting that metabolite profiles were dependent on the treatment. Notably, the ZnO25 and ZnO50 treatments exhibited a similar clustering pattern, indicating a comparable impact on the spatial distribution of synthesized metabolites in soybean roots. Given that certain metabolites exhibited analogous responses to stress, a k-means clustering algorithm was applied to further explore the accumulation patterns of DAMs in relation to the various treatments. These same 200 metabolites were subsequently categorized into nine sub-clusters based on their expression trends. The distribution of metabolites within these sub-clusters were varied, with sub-cluster 1 containing 18 metabolites, sub-cluster 2 containing 32, sub-cluster 3 containing 49, sub-cluster 4 containing 37, sub-cluster 5 containing 9, sub-cluster 6 containing 21, sub-cluster 7 containing 13, sub-cluster 8 containing 13, and sub-cluster 9 containing 8 metabolites, as depicted in Figure 4 and detailed in Table S5.

### 2.4. KEGG Pathways Analysis

**Figure 5:**
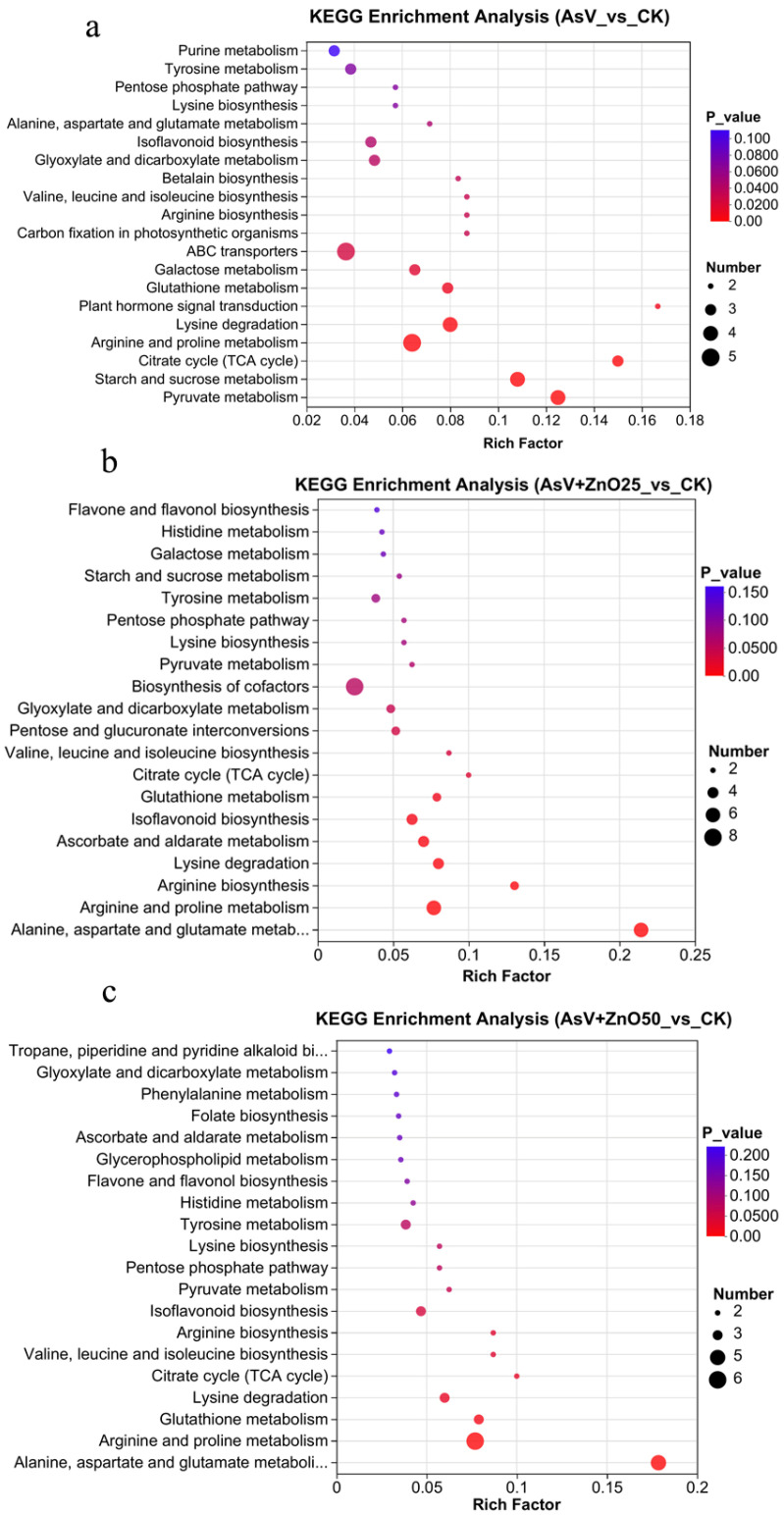
KEGG enrichment analysis of top 20 pathways of DRMs in the soybean roots: (**a**) AsV_vs_CK; (**b**) AsV + ZnO25_vs_CK; and (**c**) AsV + ZnO50_vs_CK. The size of the bubble in the figure represents the amount of enrichment to the metabolic concentration in the pathway, and the color of the bubble represents the size of the *p*-value of different enrichment significance. The treatment concentration was 25 µmol L^−1^ AsV and ZnONPs at concentrations of 25 µmol L^−1^ (2.035 mg L^−1^) and 50 µmol L^−1^ (4.07 mg L^−1^).

A KEGG (Kyoto Encyclopedia of Genes and Genomes) pathway enrichment analysis was conducted to investigate key metabolic pathways, with a significance level set at *p* < 0.05 (Figure 5a–c). Under AsV stress, metabolites were predominantly associated with arginine and proline metabolism, the tricarboxylic acid (TCA) cycle, starch and sucrose metabolism, galactose metabolism, ABC transporters, and glutathione metabolism, among other pathways (Figure 5a). In the presence of ZnO25 supplementation, metabolites were enriched in arginine and proline metabolism, arginine biosynthesis, lysine degradation, isoflavonoid biosynthesis, the TCA cycle, and ascorbate and aldarate metabolism (Figure 5b). For ZnO50 supplementation, the enriched pathways included alanine, aspartic acid, and glutamate metabolism, arginine and proline metabolism, the TCA cycle, valine, leucine, and isoleucine biosynthesis, glutathione metabolism, isoflavonoid biosynthesis, and arginine biosynthesis, among others (Figure 5c). Notably, the TCA cycle, glutathione metabolism, and arginine and proline metabolism were consistently enriched across all three treatment conditions. Importantly, the starch and sucrose metabolism pathway was exclusively enriched under AsV treatment.

## 3. Discussion 

Zinc is an essential micronutrient for plants, playing a crucial role in promoting their growth. However, its efficacy is contingent on its concentration. Furthermore, zinc significantly alleviates various forms of abiotic stress in plants, such as that induced by arsenic [12,16]. Arsenic phytotoxicity is commonly associated with disruptions of the photosynthetic apparatus, growth inhibition, and the accumulation of reactive oxygen species (ROS), leading to membrane lipid peroxidation and reduced plant biomass. Plants have developed a range of molecular regulatory mechanisms, signal transduction pathways, and metabolic alterations to tolerate and counteract arsenic toxicity. These adaptive strategies primarily involve changes in metabolic pathways, the induction of antioxidative defenses, the accumulation of osmolytes, the synthesis of phytochelatins, the production of stress hormones, and the activation of transport and sequestration systems to manage toxic ions [32]. Our previous study indicated that the application of ZnONPs modified hormonal signaling pathways and significantly regulated genes responsible for stress response, transcription factors, and transporters, thereby conferring tolerance to AsV in soybean [12]. However, the metabolic response of soybean roots to AsV toxicity and ZnONPs have not yet been fully elucidated.

Under abiotic stress, plant metabolism undergoes significant alterations. The primary objective of studying metabolic alterations during stress responses is to identify metabolites that facilitate the restoration of homeostasis and normal metabolic function [33]. Primary metabolites are particularly affected by stress, as evidenced by previous studies [34]. For instance, abiotic stress has been shown to greatly influence the primary metabolism in various plant species, such as soybean under heat and drought stress [35], maize under Pb stress [36], *Amaranthus hypochondriacus* under Cd toxicity [37], *Pteris vittata* under As toxicity [23], and rice and barley under NaCl stress [38,39]

### 3.1. The Regulation of DAMs Involved in TCA Cycle in Response to ZnONPs

**Figure 6:**
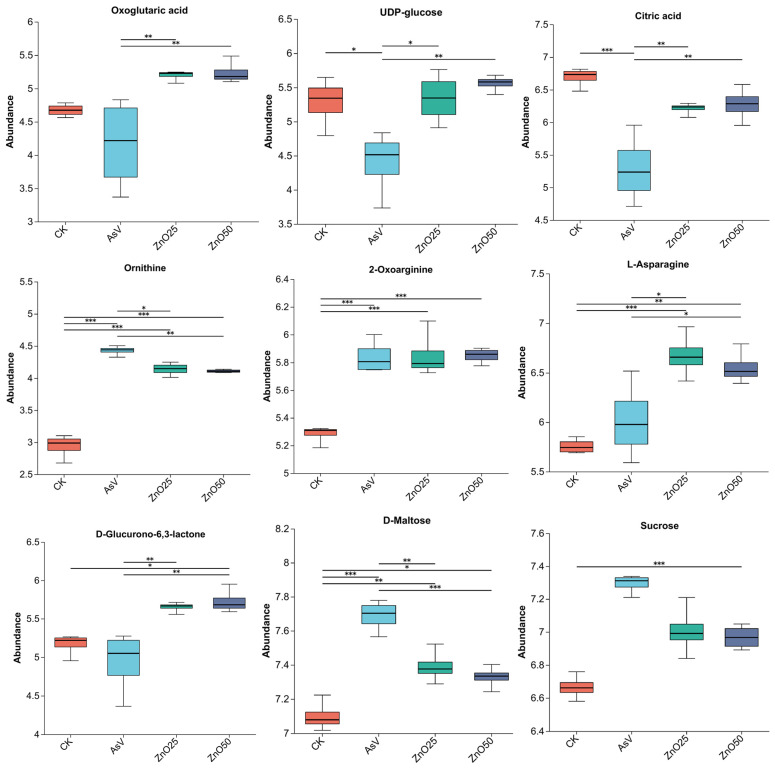
Box plots of relative abundance of some crucial metabolites in soybean roots under CK, AsV, AsV + ZnO25, and AsV + ZnO50 (*n* = 4). The X-axis represents the treatment name, and the Y-axis represents the average relative abundance of metabolites in the different treatment. The treatment concentration was 25 µmol L^−1^ AsV and ZnONPs at concentrations of 25 µmol L^−1^ (2.035 mg L^−1^) and 50 µmol L^−1^ (4.07 mg L^−1^). Asterisks (*), (**), and (***), indicate significant differences between the treatment at *p* < 0.05, *p* < 0.01 and *p* < 0.001 respectively.

The TCA cycle, also known as the Krebs cycle, is a sequence of enzymatic reactions that aerobic organisms utilize to generate ATP by oxidizing acetyl-CoA, which originates from carbohydrates, fats, and proteins [40]. In this study, the TCA pathway exhibited significant regulation across all experimental treatments compared to the control. For example, under AsV alone toxicity, a key intermediate of TCA such as citric acid, malic acid and phosphoenolpyruvic were down-regulated, whereas an elevation was observed in citric acid and oxoglutaric acid levels in AsV + ZnO50 treatment. Conversely, malic acid and phosphoenolpyruvic remained unchanged in response to ZnONP treatments (Figure 6; Table S6). As integral components of the TCA cycle, citric acid and its derivatives are essential for complex metabolic pathways that support the biosynthesis of numerous amino acids, phytohormones, secondary metabolites, and organic acids. These compounds play a critical role in alleviating various abiotic stresses, including heavy metal toxicity [41]. The reduced accumulation of citric acid indicates that the TCA cycle was down-regulated to conserve energy in response to AsV toxicity, potentially leading to decreased ATP levels and reduced plant photosynthesis and carbon fixation, which may impact soybean yield [35]. Campos et al. [42] demonstrated that in *P. calomelanos*, exposure to 30 mM As resulted in decreased levels of citric acid and other TCA intermediates such as malate, aconitate, and fumarate, which were positively correlated with plant necrosis. Similarly, As exposure inhibits the TCA cycle in non-hyperaccumulating plants due to suppressed citric acid accumulation and carbon flow, leading to reduced biomass production [43]. In contrast, As treatment was found to stimulate the TCA cycle in *P. vittata*, an As hyper-accumulator species [23]. Previous studies have shown that ZnONP application increased the levels of TCA cycle intermediates under both salinity and non-salinity stress conditions in *Sophora alopecuroides* [13]. These findings suggest that the upregulation of the TCA cycle pathway in response to ZnONPs enhances energy supply, regulates metabolism, and confers tolerance to AsV toxicity.

### 3.2. ZnONPs Modulated the Amino Acid Metabolism in Soybean Roots Under AsV Stress 

Intermediate metabolites and protectants such as amino acids play a crucial role in plant physiology [44]. Proline, for instance, functions as an osmoticum, a ROS radical scavenger, a macromolecule stabilizer, and a metal chelator under heavy metal stress, thereby conferring protection against such stressors [36,45]. Arginine, on the other hand, serves as a significant storage and transport form for organic nitrogen in plants. Beyond its role in protein synthesis, arginine is a precursor for polyamines and nitric oxide (NO) and is essential for numerous cellular and developmental processes [46] (and see the cited references). Furthermore, arginine constitutes 50% of the nitrogen (N) in the free amino acid pool within developing soybean embryos, as reported by Micallef and Shelp [47].

In this investigation, we observed that arginine and proline metabolism was markedly enriched following exposure to AsV, both independently and when compared with ZnONPs supplementation. Seven metabolites—N2-succinyl-L-ornithine, 1-pyrroline-2-carboxylic acid, 4-guanidinobutanoic acid, L-glutamate, 1-pyrroline-5-carboxylic acid, ornithine, and 2-oxoarginine—were identified as significantly enriched (Figure 6; Table S6). Notably, six of these metabolites exhibited specific and significant accumulation in response to ZnONPs treatment, whereas they were down-regulated or remained unchanged under AsV alone treatment. Notably, L-glutamate was specifically down accumulated under AsV alone. The role of amino acids in plants is pivotal for detoxification processes, encompassing the regulation of ion transport, ion chelation, and N metabolism under heavy metal stress conditions [48]. In our previous study, we found high level of proline in the As-treated soybean roots and shoots samples in response to the ZnONPs [3]. When exposed to lead stress, both maize and vetiver exhibited increased levels of amino acids in their root tissues, which positively correlated with their tolerance mechanisms [36]. Concurrently, the supplementation of ZnONPs regulated the accumulation of valine, isoleucine, and leucine—branched-chain amino acids—in soybean roots. These amino acids are biosynthesized by four enzymes and derived from the shikimate pathway. They are crucial for the production of anthocyanins, flavonoids, and lignin, as well as for protein synthesis. These aromatic secondary metabolites are necessary for non-enzymatic antioxidative defense and cell wall extensibility under heavy metal toxicity [49]. Prell et al. [50] noted that these amino acids are vital for symbiotic nitrogen fixation in leguminous plants and are essential for bacteroid development. Thus, the presence of the valine, leucine, and isoleucine pathway in our metabolite dataset suggests that ZnONPs supplementation assists in managing osmotic stress induced by AsV toxicity. Moreover, the increased levels of proline and arginine biosynthesis further support this hypothesis.

The regulation of metabolic pathways, including the aspartic acid family pathway and the TCA cycle, is essential in modulating plant physiological responses to abiotic stress. Our analysis revealed that certain amino acids within the alanine, aspartate, and glutamate pathways, such as citric acid, L-asparagine, L-glutamine, oxoglutaric acid, N-acetylaspartate, and 1-pyrroline-5-carboxylic acid exhibited reduced accumulation upon exposure to AsV (Figure 6 and Table S6). Conversely, treatment with ZnONPs led to a significant increase in the levels of these metabolites, indicating their potential involvement in conferring tolerance to AsV. This effect is attributed to the production of various metabolites by these pathways, which contribute to plant resilience against stress. These include anaplerotic reactions that maintain the TCA cycle, aspartic acid as a precursor for various protein syntheses, 4-aminobutanoic acid (GABA) for osmotic regulation, and glutamic acid for the synthesis of glutathione (GSH). Moreover, the correlation between the three types of phytochelatins (PCs) and their associated metabolites was determined [37]. The results indicated that metabolites from three pathways—valine, leucine, and isoleucine biosynthesis; alanine, aspartate, and glutamate metabolism; and arginine and proline metabolism—exhibited a strong linear correlation with PCs. This correlation can be partially attributed to the upstream and downstream relationships between these compounds. It is therefore proposed that these metabolic pathways significantly impact the synthesis of PCs, and their regulation may enhance PC levels. This is consistent with our previous findings, which demonstrated a substantial increase in PC accumulation in soybean tissues following ZnONPs treatment in As-treated plants [12]. Hence, it is confirmed that the aforementioned pathways are crucial for AsV tolerance in response to ZnONPs supplementation and warrant further investigation for the advancement of phytoremediation strategies.

### 3.3. Role of Glutathione Metabolism and Ascorbate and Aldarate Metabolism in AsV Tolerance in Response to ZnONPs

This study also identified two metabolic pathways—glutathione metabolism and ascorbate and aldarate metabolism—that exhibited up-regulation in response to zinc ZnONPs under AsV toxicity. The glutathione metabolism pathway was particularly enriched with metabolites—L-glutamate, ornithine, and 5-L-glutamyl-L-alanine—which displayed elevated accumulation exclusively under ZnONPs supplementation. Importantly, oxidized glutathione was minimally accumulated under ZnONPs treatment (Figure 6; Table S6). In contrast, oxidized glutathione and L-glutamate were accumulated in AsV alone treatment to high and low degrees, respectively. Glutathione (GSH) is crucial in plants, regulating a variety of metabolic functions such as antioxidant defense, immunity modulation, redox balance maintenance, xenobiotic detoxification, apoptosis, cell cycle regulation, cysteine storage, and fibrogenesis [38,51]. Glutamate is integral to oxidative defense mechanisms as it constitutes the fundamental component of GSH [52]. The concurrent elevation of these three amino acids underscores the critical role of GSH in AsV tolerance. Consequently, the glutathione pathway has emerged as a central area of inquiry for plants experiencing abiotic stress.

In parallel, the metabolism of ascorbate and aldarate pathway, which includes compounds such as arabinonic acid, uridine diphosphate glucose, oxoglutaric acid, and D-glucurono-6,3-lactone, has been observed to be specifically and markedly modulated in response to ZnO25 treatment, whereas under AsV treatment, this pathway was not significantly regulated. Ascorbic acid (AsA) also plays a pivotal role in alleviating arsenic stress responses and is recognized as an antioxidant that neutralizes reactive oxygen species (ROS) generated under arsenic and other abiotic stress conditions [3,53]. GSH and AsA are essential non-enzymatic antioxidants for maintaining structural integrity within plant cells. Moreover, both GSH and AsA possess the ability to reduce toxin accumulation within cells [54]. The GSH-ascorbate cycle appears to play a significant role in antioxidant defense in these species. Ascorbate and GSH serve as key cellular redox buffers, facilitating the detoxification of ROS and the transmission of redox signals [55].

In our previous investigation, the introduction of ZnO-NPs and/or selenium nanoparticles (Se-NPs) was found to stimulate the activity of enzymes within the AsA–GSH cycle, resulting in elevated levels of associated metabolites, namely, AsA and GSH [3]. Ascorbate plays a more significant role in scavenging superoxide anions (O_2_^−•^), the primary reactive oxygen species within the cell, as opposed to hydrogen peroxide (H_2_O_2_) or hydroxyl radicals (OH). It functions as a cofactor for enzymes of the Fe(II)/2-oxoglutarate-dependent dioxygenases family by reducing ferric iron (Fe(III)) to ferrous iron (Fe(II)), thereby restoring enzymatic activity. During the catalytic process, molecular oxygen (O_2_) binds to Fe(II), facilitating the transfer of one oxygen atom to 2-oxoglutarate and another to the substrate [56,57]. These metabolites could serve as key biomarkers for AsV stress tolerance in soybean. Notably, the majority of these compounds were found to increase with ZnONPs treatment, suggesting their positive contribution to arsenic tolerance.

### 3.4. Exclusively Up-Regulated Pathway Under AsV Treatment

Research has demonstrated that carbohydrate metabolism plays a crucial role in abiotic stress tolerance [58]. The carbohydrates produced via photosynthesis are vital for providing energy to cellular metabolism, as well as for regulating osmotic potential and safeguarding biomolecules [59]. The starch and sucrose metabolism pathway is exclusively up-regulated in response to AsV treatment, whereas it remains unaltered under ZnONPs exposure (Figure 5). Specifically, three compounds—sucrose, D-maltose, and levan—exhibit increased expression, while one compound, UDP-glucose, shows decreased expression in the AsV versus CK treatment (Figure 6; Table S6). Our research indicates that the elevation in sugar levels serves as a mechanism to enhance tolerance in soybean seedlings subjected to AsV-induced stress by facilitating the degradation of starch. This adaptive response is consistent with the findings of Roychoudhury et al. [60], who reported increased sugars accumulation in rice leaves under Cd toxicity, which helped stabilize the cell membrane and mitigate osmotic imbalance. Similarly, Nada et al. [61] observed an increase in sugar content in almonds under Cd stress, suggesting a defensive role. These observations imply that starch and sucrose metabolism are pivotal metabolic pathways in the response to AsV stress.

## 4. Materials and Methods

### 4.1. Experimental Design and Treatment Detail

The hydroponic experiment using the soybean genotype ZhongHuang302 involved germinating seeds in sterile vermiculite for a period of ten days. Subsequently, uniform seedlings were transplanted into 10 L pots containing modified half-strength Hoagland’s solution, as detailed previously [62]. These seedlings were cultivated under ambient temperatures ranging from 25 to 28 °C and exposed to a 15 h photoperiod with a 9 h dark cycle with 200–300 (L_250_–L_300_) μmol m^−2^ s^−1^ light intensity, supplemented by LED artificial lighting. Upon reaching the V2 growth stage (i.e., the emergence of the first two trifoliolate leaf nodes), the nutrient solutions were either maintained as is or enriched with 25 μmol L^−1^ of arsenate (Na_2_HAsO_4_) alone or in combination with ZnONPs at concentrations of 25 μmol L^−1^ (2.04 mg L^−1^) or 50 μmol L^−1^ (4.07 mg L^−1^). The control group consisted of pots receiving only the nutrient solution. These treatments were selected based on preliminary study and the detail can found in Zeeshan et al. [3]. The ZnONPs and Na_2_HAsO_4_ were procured from Sigma Aldrich, MO, USA, and utilized without further purification. The ZnONPs, with a size of 20 nm, exhibited a zeta potential ranging from −16 to 23 mV in aqueous solution, as measured by a zeta potential analyzer (NanoBrook, Brookhaven, GA, USA). Characterization of the ZnONPs was conducted using X-ray diffraction and energy dispersion spectra mapping, as outlined in Zeeshan et al. [3], whereas SEM and TEM images can be found in Figure S3. The ZnONPs stock solution, at a concentration of 4.07 mg L^−1^, was prepared in double-distilled water (ddH_2_O), and to prevent NPs aggregation and ensure their uniform suspension in the nutrient solution, the pots were routinely stirred. The pH of the nutrient solution was maintained at approximately 5.8, and the solution was refreshed twice weekly. After a ten-day treatment period, the roots were harvested, rinsed thoroughly with ddH_2_O, and were frozen in liquid nitrogen and stored at −80 °C for subsequent metabolomic analysis.

### 4.2. Metabolic Extraction, Quality Control, and Profiling 

The methodology for collecting root samples for extracting metabolites was consistent with that described by Zeeshan et al. [12]. Each treatment was replicated four times. For the extraction of metabolites from root tissues, 50 mg of fresh tissue was placed into a 2 mL centrifuge tube containing a 6 mm diameter grinding bead. The tissue was pulverized in liquid nitrogen, and metabolites were subsequently extracted by adding 400 μL of an extraction solution, which consisted of methanol and water (4:1, *v*:*v*) with an internal standard of L-2-chlorophenylalanine at a concentration of 0.02 mg/mL. The samples were ground using a Wonbio-96c frozen tissue grinder (Shanghai Wanbo Biotechnology Co., LTD, Shanghai, China) for 6 min at −10 °C and 50 Hz, followed by low-temperature ultrasonic extraction for 30 min at 5 °C and 40 kHz. Post-grinding, the samples were stored at −20 °C for 30 min then centrifuged at 13,000 g and 4 °C for 15 min. The supernatant was collected and used for LC-MS/MS analysis. To ensure system conditioning and quality control, a pooled QC sample was prepared by mixing equal volumes from all samples. These QC samples were periodically injected (every 5–15 samples) to assess the stability of the analytical process.

The LC-MS/MS analysis of the sample was performed using a Thermo Scientific UHPLC-Q Exactive HF-X system coupled with an ACQUITY HSS T3 column (100 mm × 2.1 mm internal diameter, 1.8 μm particle size; Waters, Milford, MA, USA). The analysis was conducted at Majorbio Bio-Pharm Technology Co., Ltd. (Shanghai, China), adhering to standard protocols. The mobile phase system consisted of solvent A, which was a mixture of water with 0.1% formic acid and acetonitrile (95:5, *v*/*v*); and solvent B, which was composed of 0.1% formic acid in acetonitrile, isopropanol, and water (47.5:47.5:5, *v*/*v*/*v*). The flow rate was maintained at 0.40 mL/min, and the column was operated at a temperature of 40 °C.

Mass spectrometry data acquisition was carried out using a Thermo UHPLC-Q Exactive HF-X Mass Spectrometer equipped with an electrospray ionization (ESI) source and operated in both positive and negative ionization modes. The optimal parameters were established as follows: an auxiliary gas flow rate of 13 arb, a sheath gas flow rate of 50 arbitrary units (arb), a source temperature of 425 °C, and an ion-spray voltage floating (ISVF) of 3500 V for the positive mode and −3500 V for the negative mode. The normalized collision energy was set to a range of 20–40–60 V for MS/MS experiments. The full MS resolution was set at 60,000, with an MS/MS resolution of 7500. Data were collected using the data-dependent acquisition (DDA) mode, and the detection was conducted across a mass-to-charge ratio (*m*/*z*) range of 70–1050.

### 4.3. Data Anslysis

The preprocessing of LC/MS raw data was conducted using Progenesis QI software (Waters Corporation, Milford, CT, USA), which yielded a three-dimensional data matrix in CSV format. This matrix encompassed sample details, metabolite names, and mass spectral response intensities. Subsequently, internal standard peaks and recognized false positive peaks, such as column bleed, noise, and derivatized reagent peaks, were removed from the data matrix. The process also involved deduplication and peak integration. Concurrently, metabolite identification was achieved through database searches, primarily utilizing the Metlin (https://metlin.scripps.edu/, accessed on 10 January 2022), the Human Metabolome Database (HMDB; http://www.hmdb.ca/, accessed on 10 January 2022), and the Majorbio Database.

The data matrix acquired from database searches was uploaded to the Majorbio Cloud Platform (https://cloud.majorbio.com, accessed on 10 January 2022) for subsequent analysis [63]. Initially, the data matrix underwent preprocessing, retaining at least 80% of the metabolic features detected across any sample set. Following filtration, for samples with metabolite levels below the quantification limit, the minimum metabolite value was estimated, and each metabolic signature was normalized relative to the total sum. To minimize errors associated with sample preparation and instrument instability, the peak response intensities from the sample mass spectrometry were normalized using the sum normalization method, yielding the normalized data matrix. Concurrently, variables from QC samples with a relative standard deviation (RSD) exceeding 30% were discarded, and the data were log10-transformed, resulting in the final data matrix for subsequent analyses.

The R package “ropls” (version 1.6.2) was employed to conduct principal component analysis (PCA) and orthogonal partial least squares discriminant analysis (OPLS-DA), along with a seven-cycle cross-validation to assess the model’s stability. Metabolites with a variable importance in the projection (VIP) score greater than 1 and a *p*-value less than 0.05 were identified as significantly different based on the VIP scores from the OPLS-DA model and the *p*-values from the Student’s t-test. Differential metabolites between the two groups were mapped into biochemical pathways using metabolic enrichment and pathway analysis, referencing the KEGG database (http://www.genome.jp/kegg/, accessed on 10 January 2022). These metabolites were categorized by their associated pathways or functions. Enrichment analysis was utilized to examine the presence or absence of a group of metabolites within a functional node, transitioning from the annotation of individual metabolites to that of a collective. Python’s “scipy.stats” package (https://docs.scipy.org/doc/scipy/, accessed on 10 January 2022) was utilized for enrichment analysis to identify the most pertinent biological pathways related to the experimental treatments.

## 5. Conclusions

This study elucidates the metabolic differences between soybean roots exposed to AsV alone and those treated with AsV in conjunction with ZnONPs. The introduction of ZnONPs to AsV-stressed plants significantly altered the metabolic profile of soybean roots. Notably, there was an accumulation of amino acids and their derivatives, along with enhancements in the TCA cycle, glutathione metabolism, ascorbate, and aldarate pathways, collectively contributing to the overall increase in antioxidant capacity under ZnONPs supplementation. Additionally, the concurrent increase in osmolytes and antioxidants suggests a potential role in the acquired tolerance to AsV due to ZnONPs. Similarly, the observed enhancement in starch and sucrose metabolism pathways under AsV treatment suggests a possible role for carbohydrates beyond their conventional antioxidant functions, meriting further investigation.

## Data Availability

The original contributions presented in the study are included in the article. Further inquiries can be directed to the corresponding authors.

## Supplementary Materials

The following supporting information can be downloaded at https://www.mdpi.com/article/10.3390/plants13223142/s1: Figure S1: Heatmap displaying Pearson correlation coefficients for all samples; Figure S2: UpSet Venn plot of the raw dataset from metabolomics; Figure S3: SEM and TEM images of ZnONPs; Table S1: Total ion number and identification statistics table; Table S2: List of differentially regulated metabolites (DRMs) under AsV_vs_CK treatment; Table S3: List of differentially regulated metabolites (DRMs)under AsV + ZnO25_vs_CK treatment; Table S4: List of differentially regulated metabolites (DRMs)under AsV + ZnO50_vs_CK treatment; Table S5: Hierarchical clustering algorithm to the accumulation patterns of DRMs in the soybean roots under different treatments; Table S6: List of significantly enriched metabolic pathways and metabolites play crucial role in AsV tolerance under ZnONPs supplementation.
