# Supplementary material for: Zinc Oxide Nanoparticle-Mediated Root Metabolic Reprogramming for Arsenic Tolerance in Soybean
Source: Plants (Basel). 2024 Nov 8;13(22):3142. doi: 10.3390/plants13223142 (PMC11597289; doi:10.3390/plants13223142)
Supplement: Supplementary file 1 [file plants-13-03142-s001.zip › Supplementary file2.pdf]

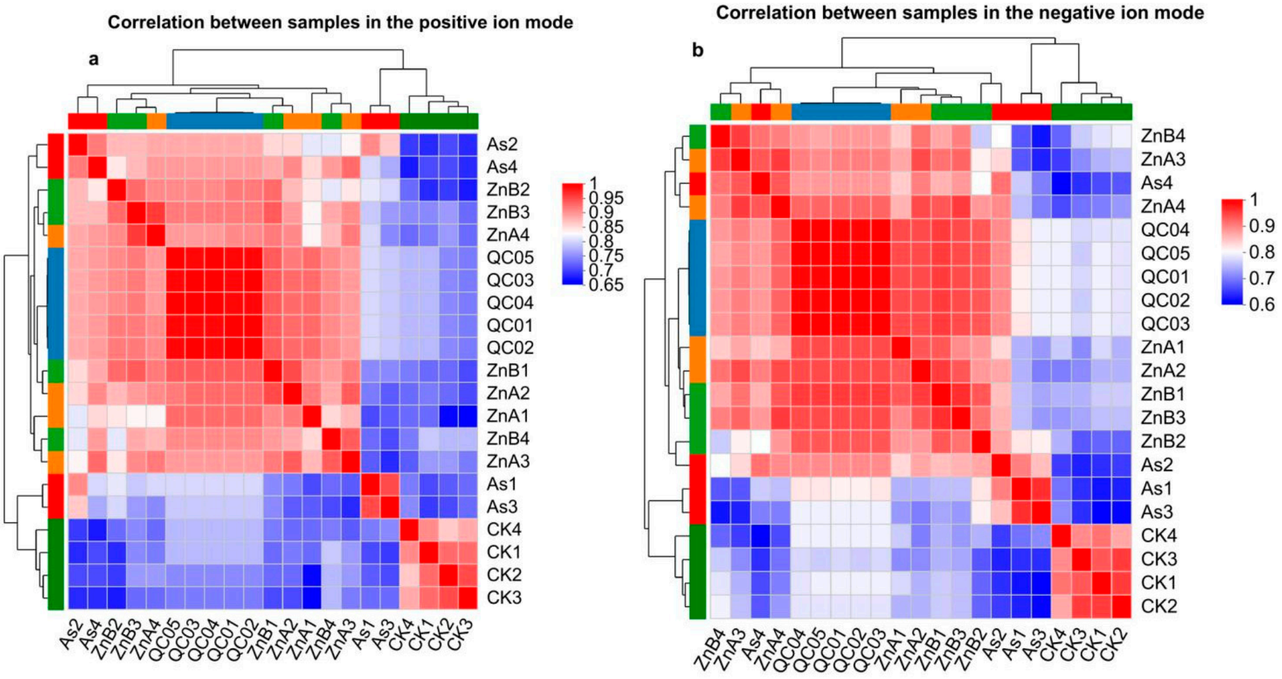

Figure S1. Heatmap displaying Pearson correlation coefficients for all samples. a) Positive ion mode b) Negative ion mode. The right and bottom sides of the figure list the sample names. Each cell in the heatmap represents the correlation between two samples. Different colors indicate the relative magnitude of the correlation coefficient, and the length of the clustered branches signifies the relative distance between samples, with those closer together on the same branch being more similar.

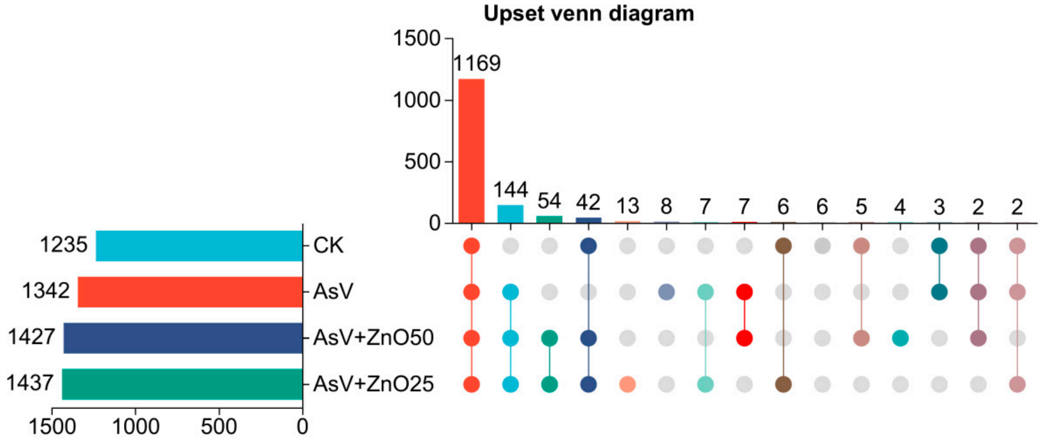

Figure S2. UpSet venn plot of the raw dataset from metabolomics. The histogram in the lower left corner represents the count of elements in each group. The bar chart on the right shows the statistical results of the number of elements after the intersection of various groups. The single point below indicates the elements unique to a group, and the line connecting the points represents the intersection between groups.

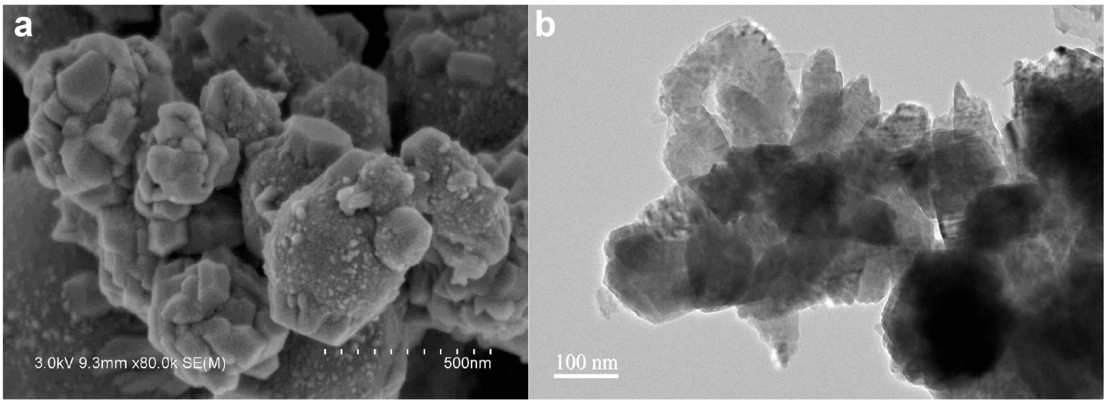

Figure S3. Characterization of ZnoNPs using SEM (a) TEM (b) showing the morphology of the NPs.
